# Frequency and risk of SARS-CoV-2 reinfections in Norway: a nation-wide study, February 2020 to January 2022

**DOI:** 10.1186/s12889-024-17695-8

**Published:** 2024-01-15

**Authors:** Håkon Bøås, Margrethe Larsdatter Storm, German Tapia, Anja Bråthen Kristoffersen, Astrid Louise Løvlie, Ketil Størdal, Trude Marie Lyngstad, Karoline Bragstad, Olav Hungnes, Lamprini Veneti

**Affiliations:** 1 Department of Infection Control and Vaccines, Norwegian Institute of Public Health, Lovisenberggata 8, 0456 Oslo, Norway; 2 Department of Infectious Disease Registries, Norwegian Institute of Public Health, Oslo, Norway; 3 Department of Chronic Diseases, Norwegian Institute of Public Health, Oslo, Norway; 4 Department of Method Development and Analytics, Norwegian Institute of Public Health, Oslo, Norway; 5 Institute of Clinical Medicine, University of Oslo, Oslo, Norway; 6 Division of Pediatric and Adolescent Medicine, Oslo University Hospital, Oslo, Norway; 7 Department of Infection Control and Preparedness, Norwegian Institute of Public Health, Oslo, Norway; 8 Department of Virology, Norwegian Institute of Public Health, Oslo, Norway

**Keywords:** Reinfections, Norway, SARS-CoV-2, COVID-19, Omicron

## Abstract

**Background:**

SARS-CoV-2 reinfection rates have been shown to vary depending on the circulating variant, vaccination status and background immunity, as well as the time interval used to identify reinfections. This study describes the frequency of SARS-CoV-2 reinfections in Norway using different time intervals and assesses potential factors that could impact the risk of reinfections during the different variant waves.

**Methods:**

We used linked individual-level data from national registries to conduct a retrospective cohort study including all cases with a positive test for SARS-CoV-2 from February 2020 to January 2022. Time intervals of 30, 60, 90 or 180 days between positive tests were used to define potential reinfections. A multivariable Cox regression model was used to assess the risk of reinfection in terms of variants adjusting for vaccination status, demographic factors, and underlying comorbidities.

**Results:**

The reinfection rate varied between 0.2%, 0.6% and 5.9% during the Alpha, Delta and early Omicron waves, respectively. In the multivariable model, younger age groups were associated with a higher risk of reinfection compared to older age groups, whereas vaccination was associated with protection against reinfection. Moreover, the risk of reinfection followed a pattern similar to risk of first infection. Individuals infected early in the pandemic had higher risk of reinfection than individuals infected in more recent waves.

**Conclusions:**

Reinfections increased markedly during the Omicron wave. Younger individuals, and primary infections during earlier waves were associated with an increased reinfection risk compared to primary infections during more recent waves, whereas vaccination was a protective factor. Our results highlight the importance of age and post infection waning immunity and are relevant when evaluating vaccination polices.

**Supplementary Information:**

The online version contains supplementary material available at 10.1186/s12889-024-17695-8.

## Background

In August 2020, the first case of SARS-CoV-2 reinfection was described in Hong Kong, followed by numerous cases worldwide [1, 2]. Reinfections, described as a person infected with an agent, recovered, and then infected again at a later time, could be caused by either the same variant or a new variant of the same agent [3]. The reinfection rate of SARS-CoV-2 has been reported to be between less than 0.5% to more than 5% depending on the dominant variant at the time of investigation, duration of the study, as well as the country, population studied, vaccination coverage and background immunity [2, 4–7]. The European Center for Disease Control (ECDC) currently defines a suspected COVID-19 reinfection as a positive PCR or rapid antigen test ≥60 days following a previous positive PCR, rapid antigen test or serology [8]. In contrast, the WHO case definition proposes at least 90 days between the episodes or, alternatively, genomic evidence of different lineages in the two episodes regardless of time interval to be considered a reinfection [9]. Countries have also used other intervals and criteria for reporting suspected reinfections [8]. In a survey conducted by ECDC in 2021, 13 European countries reported having a case definition, however the time interval between episodes ranged from 45-90 days among the countries, where the majority of countries used 90 days. Five countries had also included symptom-free periods in their case definitions. Although the case definitions were similar, they were not standardized. Thus, there is a need for an assessment of the intervals used to identify reinfections to be able to make comparisons across different countries and regions.

The first case of SARS-CoV-2 in Norway was detected on February 26 2020 [10]. Testing criteria and recommendations have changed throughout the pandemic in Norway. Up to May 2020, there was limited availability of SARS-CoV-2 testing and only selected groups were tested. Following this period, test capacity was strengthened, and all individuals that had any respiratory symptom were recommended to get tested for SARS-CoV-2 [11]. By the end of 2021 testing was further scaled up with the introduction of rapid antigen tests, which also included self-administered antigen tests. Test activity has since remained high, until recommendations were eased and restrictions lifted after the end of January 2022 [12].

**Fig. 1:**
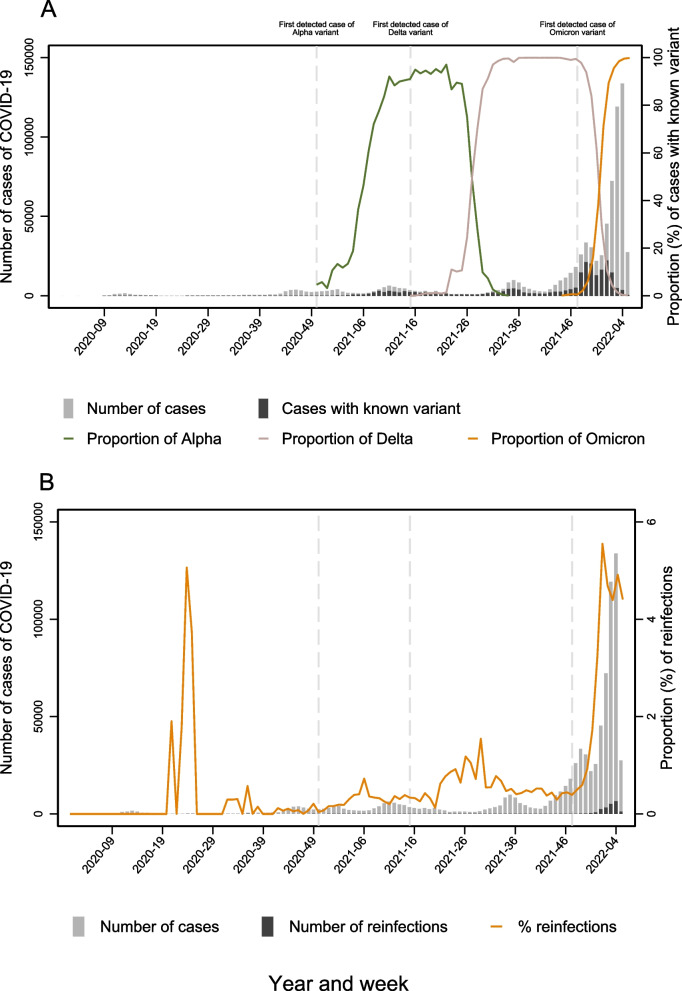
Distribution of primary and secondary COVID-19 infections in Norway from February 2020–January 2022, using a minimum interval of 60 days between re-infections, or having two results of different variant. **A** Number of COVID-19 reported cases and cases with known variant by week of sampling. The proportion of Alpha, Delta and Omicron cases presented on the secondary axis. **B** Number of reported COVID-19 cases and reinfections by week of sampling. The proportion of reinfections out of reported cases are presented on the secondary axis

The emergence of the Alpha variant raised concerns about its potential to be more transmissible or escape previously acquired immunity, resulting in increased variant surveillance. In Norway, the Alpha variant was first detected in December 2020, followed by the Delta [13], and Omicron [14] waves (Fig. 1). In Norway, cases were counted as reinfections if there was a positive PCR result 90 days following a positive PCR test from 24.03.2020, and 180 days between episodes from 01.07.2021. However, as new variants emerged, the interval was changed to 60 days from 21.01.2022, in accordance with the ECDC definition. Thus, there is a need to describe the ability of the different intervals to identify reinfections and the impact of implementing these intervals in national surveillance systems as well as assessing potential factors that could impact on the risk of reinfection.

## Methods

### Aim and data sources

The aim of this study was to describe the frequency of SARS-CoV-2 reinfections in Norway during 2020-2022 using different time intervals between infections, as well as assessing the risk of SARS-CoV-2 reinfections in terms of variants, vaccination status, demographic characteristics, and underlying comorbidities.

The data was retrieved from the Norwegian national preparedness registry for COVID-19 (BeredtC19) that covers the entire Norwegian population and contains individual level data on demographics, results of laboratory testing, vaccinations, and diagnoses from primary and specialist health services. The data are reported from central health registries, national clinical registries, and national administrative registries [15] and is linkable by a unique national identity number for all Norwegian citizens, as well as individuals born or permanently residing in Norway. We included data on positive SARS-CoV-2 tests from the MSIS laboratory database, which receives SARS-CoV-2 test results from all Norwegian microbiology laboratories and testing stations (PCR and antigen tests only, self-administered antigen tests are not registered). It is mandatory for all Norwegian microbiology laboratories to report all laboratory results, both positive and negative test results, to the MSIS laboratory database. The laboratory results are electronically reported using the National Laboratory Classification System. The MSIS laboratory database also provided data on SARS-CoV-2 variants which was reported by the Norwegian microbiology laboratories. Further details regarding the variant surveillance in Norway are described on Norwegian Institute of Public Health’s webpage [16]. Data on comorbidities were based on The Norwegian Patient Registry (NPR) and ICPC-2 codes from the Norwegian Control and Payment of Health Reimbursements Database (KUHR) as outlined previously [17], while COVID-19 vaccinations were retrieved from the Norwegian Immunization Registry, SYSVAK, and demographic variables (sex, age, county and country of birth) were from the National Population Register.

### Study design

We conducted a register based study using individual level data for the period 26 February 2020 to 31 January 2022. In these analyses, we included all cases with a positive PCR or antigen test for SARS-CoV-2 among individuals with an available national identity number. In order to assess the risk of reinfections during periods when different variants were dominant, we conducted separate cohort studies per variant wave. The definitions and methods used are clarified below.

#### SARS-CoV-2 infections and reinfections

A SARS-CoV-2 infection was defined as a person having a positive SARS-CoV-2 PCR test or antigen test registered in the MSIS laboratory database. For the primary infection, the date of the first positive test was used as time of infection. Potential reinfections were defined as, a positive PCR or rapid antigen test using intervals of minimum 30, 60, 90 and 180 days following a previous positive test. If there were several positive tests within the given time interval, these were considered as belonging to the first infection.

#### Variant waves

Using virus variant data from the MSIS laboratory database and the date of positive test, we defined variant-waves as periods when one variant was accounting for ≥ 90% of the tests that had been PCR screened or whole genome sequenced, allowing for temporary fluctuations (maximum 2 weeks) when the percentage of the dominant variant was allowed to drop down to ≥ 88%.

We defined three dominant variant-waves: The Alpha wave during weeks 11-22/2021, the Delta wave during weeks 30-49/2021 and the early Omicron wave (mainly BA.1) from week 2/2022 to the end of the study period (31 January 2022). From week 53/2020 until the beginning of the Delta wave, more than 10% of screened/whole genome sequenced cases were the Alpha variant. Prior to this, all cases that were not ascertained to be the Alpha variant, were assigned to the group “Pre-Alpha variants” (Fig. 1).

#### Vaccination status

In Norway, for the duration of this study, the mRNA vaccines Comirnaty (BNT162b2, BioNTech-Pfizer) and Spikevax® (mRNA-1273, Moderna) was primarily used. The adenoviral vector-based Vaxzevria (AstraZeneca) and Jcovden (Janssen-Cilag International NV) were used to a limited extent during 2021, until theses vaccines were suspended in Norway when concerns were raised about the increased risk of cerebral venous sinus thrombosis after vaccination [18] . The details of the vaccination program in Norway for adults and adolescents are given in detail in previous published work [19, 20]. Following the official definitions used for counting number of doses [21], we defined vaccine status using data on number of doses and date of vaccination recorded in the Norwegian Immunisation Registry (SYSVAK) as:

- Unvaccinated that have not received any COVID-19 vaccine
- Vaccinated with one dose of a COVID-19 vaccine <21 days prior
- Vaccinated with one dose of a COVID-19 vaccine >=21 days prior
- Completed primary vaccination series with two doses of a mRNA COVID-19 vaccine 7-179 days after second vaccine dose, or 21 days after an initial dose of Jcovden
- Completed primary vaccination series with two doses of a mRNA COVID-19 vaccine ≥180 days after second vaccine dose, or ≥180 days after an initial dose of Jcovden
- Vaccinated with three doses of a COVID-19 vaccine. Individuals were considered as vaccinated with three doses if the third dose was received at least 7 days prior.

#### Underlying comorbidities with increased risk of severe COVID-19

Individuals with underlying comorbidities that cause an increased risk of severe COVID-19 have been prioritized for vaccination [22]. We categorized cases into three groups: i) no underlying comorbidities, ii) medium risk comorbidity and iii) high risk comorbidity, as described elsewhere [17].

#### Statistical analysis

##### Description of reinfections

We described numbers and proportions of cases reported to MSIS by different characteristics during the study period, distinguishing primary infections and reinfections using different time intervals; 30, 60, 90 or 180 days between positive tests.

##### Risk of reinfection by variant wave

In order to assess the risk of reinfection, we conducted separate cohort studies for each variant wave. At the start of each cohort study/wave, the individuals previously infected once were included and were followed up (being at risk for reinfection) until the end of the wave. The outcome of interest was being reinfected (once) using as time interval ≥60 days since a previous positive test. Data were censored at the end of follow-up, reinfection or death. The variables we considered as exposures and that were taken into account in our analyses were sex, age, county of residence, country of birth, underlying comorbidities, vaccination status (determined daily throughout the waves) and time period of previous SARS-CoV-2 infection.

To assess the association between covariates and risk of reinfection during the different variant waves we calculated hazard ratios (HR) with 95% confidence intervals (CI) using a Cox proportional hazards model on a calendar time scale, and adjusted hazard ratios (aHR) using a stratified multivariable Cox proportional hazards model. The underlying time scale was calendar time based on sampling date, with entry at the start of each wave. We adjusted the analysis by sex, age, underlying comorbidities, vaccination status, and previous SARS-CoV-2 infection, and stratified by county of residence (11 levels) and country of birth (3 levels). We chose to adjust for vaccination and not conduct separate analyses, in order to assess the impact of vaccination without complicating the analyses. Some individuals could have been vaccinated before the start of the wave (before or after being infected once), and some could be vaccinated at different points (with one or more doses as well) of the wave before or after their reinfection. Different analyses, with different set up, should be planned if we want to explore exposures when having two distinct outcomes, reinfection (only infected before) or breakthrough infection (infection and vaccination before). In our study, our estimates are for these outcomes combined.

Proportionality was assessed using Log–log plots of survival (not shown) and found to be satisfactory. Participants were followed until endpoint, death, emigration, or end of the respective wave.

We should note that for the analyses to assess the risk off reinfection by different characteristics, we excluded 67 individuals that had received more than three vaccine doses by January 2022 as the numbers were too small to allow any comparisons [23]. Moreover, we excluded individuals with unknown county of residence as well as individuals with no reported infection prior to the variant wave of interest.

#### Sensitivity and exploratory analyses

As testing against SARS-CoV-2 was not readily available without a physician’s referral until 12 August 2020 [11], a sensitivity analysis was conducted excluding all individuals with the first infection before this date if they did not have a secondary infection before the start of the wave studied.

As part of an exploratory analysis, an additional Cox proportional hazard model was constructed where each variable in turn was included in a multivariate model with the previous episode of SARS-CoV-2 infection, stratifying for all other variables. For the variable previous episode of SARS-CoV-2 infection, sex was included in the multivariate model, stratifying for all other variables. Furthermore, a multivariate random-effects logit model was constructed, including sex, age group, risk group, vaccine status, previous episode of SARS-CoV-2 infection as independent variables (Additional files 1, 2 and 3).

Statistical analysis was performed in Stata version 17 (Stata Corporation, College Station, Texas, US).

## Results

From 26 February 2020 to 31 January 2022, 768 755 individuals were reported to have tested positive for SARS-CoV-2 in Norway. Among these, 683 121 (89%) had tested positive for SARS-CoV-2 once, 85 420 (11%) were registered with 2–5 positive tests, whereas 214 (0.03%) were reported to have more than 5 positive tests. We defined potential reinfections, using time intervals of 30, 60, 90 or 180 days between positive tests and the distributions of reinfections are presented in Table 1. As expected, the number of potential reinfections identified decreased as longer time intervals were applied, ranging from 23 879 (30 days) to 13 960 (180 days). This corresponds to an overall reinfection rate ranging from 1.8–3.1% of all infections (Table 1). Similarly, the number of reinfections where both infections occurred within the same variant wave decreased with increasing time intervals, with numbers being generally low, ranging from 333 to 2 (Table 2). In accordance with the ECDC surveillance case definition for suspected reinfection, the 60 days interval was used in the remaining part of the study.

**Table 1:** Distribution of individuals with one or more SARS-CoV-2 infections in MSIS and MSIS laboratory database

	**30-day interval, n (%)**			**60-day interval, n (%)**			**90-day interval, n (%)**			**180-day interval, n (%)**		
**Total number of infected individuals**	768755 (100)			768755 (100)			768755 (100)			768755 (100)		
**Infected once**	744876 (96.9)			747106 (97.2)			748959 (97.4)			754795 (98.2)		
**Reinfected**	23 879 (3.1)			21 649 (2.8)			19 796 (2.6)			13960 (1.8)		
**One reinfection**	23739 (3.1)			21582 (2.8)			19763 (2.6)			13954 (1.8)		
**Two reinfections**	138 (0.02)			66 (0.01)			33 (<0.00)			6 (<0.00)		
**Three reinfections**	2 (<0.00)			1 (<0.00)			-			-		
**Weeks between 1 and 2 infections, 25, 50 and 75 percentiles**	**p25**	**median**	**p75**	**p25**	**median**	**p75**	**p25**	**median**	**p75**	**p25**	**median**	**p75**
**Alpha**	6	10	18	11	17	24	17	21	29	29	35	51
**Delta**	20	36	47	27	38	49	30	39	50	35	42	52
**Omicron**	19	37	51	20	40	53	22	42	54	41	46	59
**All variants**	18	36	50	20	39	52	22	41	54	40	46	58

Distribution of individuals reported with one or more SARS-CoV-2 infections using 30-, 60-, 90- and 180-days intervals since previous infection, February 2020 to January 2022, Norway (*N*=768755). The 25^th^ percentile (p25), median and 75^th^ percentile (p75) of the number of weeks between first and second infection using 30-, 60-, 90- and 180-days intervals to define reinfections

**Table 2:** Number of possible reinfections in MSIS and MSIS laboratory database within a variant wave

	**30 days**	**60 days**	**90 days**	**180 days**
**Alpha : Alpha**	115	23	0	0
**Delta : Delta**	205	89	44	2
**Omicron : Omicron**	13	1	-	-
**Total**	333	113	44	2

Screening or sequencing results from both the first infection and the subsequent reinfection was available for 7.1% (*n*=1544) of all suspected reinfections (Table 3). When using the 60-day interval to identify reinfections, allowing for shorter intervals if information about strain were available for both first infection and reinfection, 18% (*n*=3892) of the 21 649 potential reinfections had information from variant screening or sequencing. The median time between the first infection and first reinfection was 39 weeks (interquartile range: 32 [50-20]). Omicron, primarily BA.1 with some BA.2, accounted for 85.4% (*n*=3324) of these reinfections confirmed by sequencing or screening (Table 3). Among all suspected reinfections, 86% (*n*=18 576) were assigned to a variant wave, 80% (*n*=17 340) occurred during the Omicron wave, 5% (*n*=1 018) occurred during the Delta wave and 14% (*n*=3 073) were in between waves (Fig. 1 and Table 3).

**Table 3:** Number of possible reinfections in the MSIS laboratory database with divergent variant results

	**Variant**	**First infection**	**Reinfections N (%)**					**Total N with reinfections**
		**N (%)**	**Alpha (%)**	**Delta (%)**	**Omicron (%)**	**Other variants (%)**	**Not variant screened (%)**	**N (%)**
**Screened and sequenced variants**	Alpha	37448 (100)	0 (0)	93 (0.2)	698 (1.9)	0 (0)	3409 (9.1)	4200 (11.2)
	Delta	119566 (100)	0 (0)	2 (<0.1)	543 (0.5)	0 (0)	3150 (2.6)	3695 (3.1)
	Omicron	61615 (100)	0 (0)	0 (0)	0 (0)	0 (0)	1 (<0.1)	1 (<0.1)
	Other variants	9648 (100)	5 (0.1)	40 (0.4)	162 (1.7)	1 (<0.1)	865 (9.0)	1073 (11.1)
	Not variant screened	540478 (100)	29 (<0.1)	375 (0.1)	1921 (0.4)	23 (<0.1)	10332 (1.9)	12680 (2.3)
	**TOTAL**	**768755 (100)**	**34 (<0.1)**	**510 (0.1)**	**3324 (0.4)**	**24 (<0.1)**	**17757 (2.3)**	**21649 (2.8)**
**Variant waves including screened and sequenced variants**	**Variant**	**N (%)**	**Alpha (%)**	**Delta (%)**	**Omicron (%)**	**Other variants (%)**	**Not in wave or variant screened (%)**	**N (%)**
	Alpha	53962 (100)	23 (<0.1)	327 (0.6)	4958 (9.2)	1 (<0.1)	825 (1.5)	6134 (11.4)
	Delta	215670 (100)	0 (-)	89 (<0.1)	6289 (2.9)	1 (<0.1)	994 (0.5)	7373 (3.4)
	Omicron	349782 (100)	0 (-)	0 (-)	1 (<0.1)	0 (-)	0 (-)	1 (<0.1)
	Other variants	52262	95 (0.2)	414 (0.8)	4284 (8.2)	60 (<0.1)	898 (1.7)	5751 (11.0)
	Not in wave or variant screened	97079 (100)	34 (<0.1)	188 (0.2)	1808 (1.9)	4 (<0.1)	356 (0.4)	2390 (2.5)
	**TOTAL**	**768755 (100)**	**152 (<0.1)**	**1018 (0.1)**	**17340 (2.3)**	**66 (<0.1)**	**3073 (0.4)**	**21649 (2.8)**

Number of possible reinfections with divergent variant results based on Whole Genome Sequencing or variant screening or variant waves, primary and secondary infection, using a minimum interval of 60 days between re-infections, or having two results of different variant

Considering individuals with <60 days since a previous infection as not at risk for reinfection, a total of 75 986 individuals were at risk of SARS-CoV-2 reinfection during the Alpha wave, 130 048 individuals were registered in the Delta wave while 258 107 in the early Omicron wave (10 January–31 January 2022). Among individuals at risk of reinfection, 5.9% (*n*=15 151) were registered with a reinfection during the early Omicron wave, compared to only 0.6% during the Delta wave and 0.2% during the Alpha wave. The proportion of men and women was similar (47.8%–48.9% women). Women had slightly increased risk of reinfection with Omicron (6.2% in women vs. 5.6% in men, aHR = 1.15; 95% CI 1.11–1.18; *p*<0.01) which was not observed for reinfections with other variants. Younger age groups had a higher risk of reinfection during the early Omicron wave compared to the reference group of 30–44 year-olds, with the highest risk among 12–17 year-olds (aHR = 1.67; 95% CI 1.58–1.76; *p*<0.01). The lowest risk of reinfection was among the 75 year-olds and older age group (aHR = 0.11; 95% CI 0.08–0.16; *p*<0.01). The reduced risk of infection among the older age groups was also found during the Delta wave. Having medium or high-risk comorbidities seemed to confer a reduced risk of reinfections with Omicron compared to those without comorbidities in univariate analysis. However, in the multivariate analysis only the high-risk comorbidity group had a borderline significant reduced risk of reinfections for Omicron, while the medium-risk group had a slightly increased risk (Table 4), which was not readily observed for reinfections with other variants.

**Table 4:** Characteristics of SARS-CoV-2 reinfections during the Omicron wave, using a 60-days interval between cases

	**Previously infected individuals at the start of the Omicron wave n (%)**	**Reinfections n(%)**	**Hazard ratio**	Adjusted Hazard ratio^*^	Adjusted *P*-value^*^
**Sex**					
Male	131903 (51.1)	7367 (5.6)	Ref.	Ref.	-
Female	126204 (48.9)	7784 (6.2)	1.11 (1.08-1.15)	1.15 (1.11-1.18)	<0.001
**Age (in years)**					
0-11	40110 (15.5)	2972 (7.4)	1.46 (1.39-1.54)	1.08 (1.01-1.15)	0.017
12-17	39250 (15.2)	4080 (10.4)	1.92 (1.84-2.02)	1.67 (1.58-1.76)	<0.001
18-29	52590 (20.4)	3480 (6.6)	1.17 (1.11-1.22)	1.15 (1.10-1.21)	<0.001
30-44	56581 (21.9)	3059 (5.4)	Ref.	Ref.	-
45-54	32985 (12.8)	1117 (3.4)	0.62 (0.58-0.66)	0.72 (0.67-0.77)	<0.001
55-64	19229 (7.5)	340 (1.8)	0.32 (0.29-0.36)	0.40 (0.36-0.45)	<0.001
65-74	9799 (3.8)	70 (0.7)	0.13 (0.11-0.17)	0.20 (0.16-0.25)	<0.001
>=75	7563 (2.9)	33 (0.4)	0.08 (0.05-0.11)	0.11 (0.08-0.16)	<0.001
**County**					
Agder	10035 (3.9)	508 (5.1)	Ref.	-	-
Innlandet	10805 (4.2)	413 (3.8)	0.73 (0.64-0.84)		
Møre og Romsdal	5789 (2.2)	114 (2.0)	0.39 (0.32-0.48)		
Nordland	5287 (2.0)	112 (2.1)	0.42 (0.34-0.51)		
Oslo	67501 (26.1)	5543 (8.2)	1.62 (1.48-1.77)		
Rogaland	14441 (5.6)	483 (3.3)	0.66 (0.58-0.74)		
Troms og Finnmark	9278 (3.6)	148 (1.6)	0.32 (0.26-0.38)		
Trøndelag	16329 (6.3)	669 (4.1)	0.81 (0.73-0.91)		
Vestfold og Telemark	15347 (6.0)	763 (5.0)	1.00 (0.89-1.12)		
Vestland	21439 (8.3)	840 (3.9)	0.79 (0.71-0.88)		
Viken	81856 (31.7)	5558 (6.8)	1.34 (1.23-1.47)		
**Country of birth**					
Foreign	74037 (28.7)	4563 (6.2)	Ref.	-	-
Norway	179856 (69.7)	10568 (5.9)	0.99 (0.95-1.02)		
Unknown	4214 (1.6)	20 (0.5)	0.08 (0.05-0.12)		
**Risk group**					
No comorbidity	224701 (87.1)	13796 (6.1)	Ref.	Ref.	-
Medium risk comorbidity	30137 (11.7)	1286 (4.3)	0.69 (0.65-0.73)	1.10 (1.03-1.16)	0.002
High risk comorbidity	3269 (1.3)	69 (2.1)	0.34 (0.27-0.43)	0.78 (0.62-0.99)	0.041
**Vaccine status**					
Unvaccinated	93857 (36.4)	7971 (8.5)	Ref.	Ref.	-
Vaccinated with one dose <21 days earlier	2635 (1.0)	98 (3.7)	0.42 (0.34-0.51)	0.38 (0.31-0.46)	<0.001
Vaccinated with one dose >=21 days earlier	58983 (22.9)	4778 (8.1)	0.78 (0.75-0.81)	0.72 (0.69-0.75)	<0.001
Maximum of two doses 7-179 days prior	69593 (27.0)	1880 (2.7)	0.33 (0.31-0.35)	0.40 (0.38-0.42)	<0.001
Maximum of two doses ≥180 days prior	24572 (9.5)	292 (1.2)	0.16 (0.14-0.18)	0.37 (0.32-0.41)	<0.001
Three doses	8467 (3.3)	132 (1.6)	0.21 (0.17-0.25)	0.40 (0.33-0.48)	<0.001
**Most recent infection prior to Omicron wave**					
Pre-alpha infection	42558 (16.5)	3012 (7.1)	Ref.	Ref.	-
Inter-wave pre-alpha/Alpha	30757 (11.9)	2423 (7.9)	1.12 (1.06-1.18)	0.93 (0.88-0.98)	0.006
Alpha wave infection	42321 (16.4)	3406 (8.1)	1.14 (1.09-1.20)	0.87 (0.83-0.91)	<0.001
Inter-wave Alpha/Delta	10425 (4.0)	693 (6.7)	0.93 (0.86-1.02)	0.83 (0.76-0.90)	<0.001
Delta wave infection	132046 (51.2)	5617 (4.3)	0.77 (0.74-0.81)	0.56 (0.54-0.59)	<0.001

Characteristics of SARS-CoV-2 reinfection cases during the Omicron wave, using a 60-days interval between cases. Hazard ratio estimates for reinfection using stratified Cox regression model in Norway 26 February 2020 - 31 January 2022 (*n* = 258 107)

^*^Sex, age group, risk group, vaccine status, the most recent infection prior to the Omicron wave was included in a multivariate model, stratifying for county of residence and country of birth

The risk of reinfection during the early Omicron wave varied between the counties. The highest percentage of reinfections was found in Oslo and the neighboring county Viken (8.2% and 6.8% respectively for the Omicron wave).

For the Omicron and Delta waves we did not find any differences in risk of reinfection between individuals who were born in Norway compared to people born abroad. Only 135 (0.2%) reinfections were identified among the 75 986 individuals infected before the end of the Alpha wave, making it difficult to draw conclusions about this wave. However, during the Alpha wave, those born in Norway had a reduced risk of reinfections compared to those born outside of Norway (HR 0.58 95% CI 0.41-0.82, *p*<0.01) (Additional file 4).

Vaccinated individuals had a reduced risk of reinfection compared to unvaccinated. There seemed to be little difference between those vaccinated with two or three doses, while those vaccinated with only one dose had an intermediate protection of reinfection. Having had a previous infection during the more recent waves was associated with a lower risk of reinfections compared to having a previous infection during the earlier stages of the pandemic (Table 4). Neither excluding infections from before 12 August 2020 (not shown), or stratifying on individual number SARS-CoV-2 test events (Additional file 5), had an impact on the results.

## Discussion

In this study we examined the rate of potential reinfections of SARS-CoV-2 in Norway from 2020 to early 2022, during the Alpha, Delta and early Omicron waves while exploring the use of different detection time interval criteria. Notably, during the early Omicron period, both infections and reinfections surged in Norway (Fig. 1B, Table 3), aligning with reports from other countries [5, 6, 24–26]. This increase in infections and reinfections could be attributed to enhanced infectivity [27], the immune escaping features of the Omicron variant [28], breakthrough infections among previously vaccinated people [29] and post infection waning immunity among people with previous infection [30]. These factors have been widely known and previously discussed in published reports [31]. Our study assessed the risk of SARS-CoV-2 reinfections and identified variations among population sub-groups.

Previous studies have generally used one specific time interval to define potential reinfections, with most using a 90-day minimum interval between episodes [6, 24–26, 32, 33]. In our analysis, we compared reinfections using time intervals of 30, 60, 90 or 180 days between positive tests, and we observed that the distribution of reinfection frequency did not substantially differ for intervals ≤ 90 days (Table 1). We should note that since the variant waves were defined as periods when the dominant variant was found in minimum 90% of the screened or sequenced infections, some of these reinfections within the same variant wave might be with different variants. On the other hand, studies have also shown that some individuals have persistent infection or viral shedding up to three months after an infection [9, 34]. To mitigate the possibility misclassifying persistent infections as reinfections, we chose the 60-day cut-off to define potential reinfections, consistent with ECDC’s guidelines/definition [8]. Using a 60-day cut off to define reinfections, the reinfection rate ranged from 0.2% during the Alpha wave to 0.6% during the Delta wave and peaked at 5.9% during the early Omicron wave (Table 4, Table 5, Additional file 4). Previous studies have reported a range of reinfection rates of SARS-CoV-2 from less than 0.5% to above 5% [2, 4–7]. Diverse reinfection rates reported globally could be due to differences in case definitions for reinfections, study timing, and duration considering different variants and vaccine availabilities, as well as differences in testing activity and infection pressure. It is important to acknowledge that this study did not directly account for changes in infection pressure and testing which is a limitation. To adjust for test activity, a secondary analysis that stratified on individual test activity was performed (Additional file 5). Stratifying for test activity did not affect the results of the study. The considerably higher infection pressure during the early Omicron wave is in itself expected to have increased the likelihood of reinfection. Infection pressure is thought to be correlated with age and county. While our study adjusted for age and county in a multivariable Cox-regression model, accounting for potential biases, the complexity of infection pressure and testing nuances during the Omicron wave demands cautious interpretation. We should note that the rates of reinfections may have been underestimated in our study and other studies, as the existing surveillance systems could not detect all the asymptomatic infections. As the Omicron variants have been reported to be less severe and more often asymptomatic than Alpha and Delta [35], the underestimation of the reinfections due to asymptomatic cases could be higher during the early Omicron wave compared to preceding waves. Also, the reinfection rate for Omicron could be further underestimated, as the Omicron period in our study was restricted to the early phase of the Omicron occurrence. Lastly, self-administered antigen tests are not reported to the national surveillance system in Norway. This could cause further underestimations of the number of reinfections. Therefore, our results should be interpreted with caution and restricted for the period included.

**Table 5:** Characteristics of SARS-CoV-2 reinfections during the Delta wave, using a 60-day interval between cases

	**Previously infected individuals at the start of the Delta wave n (%)**	**Reinfections n(%)**	**Hazard ratio**	Adjusted Hazard ratio^*^	Adjusted *P-*value^*^
**Sex**					
Male	67840 (52.2)	423 (0.6)	Ref.	Ref.	-
Female	62208 (47.8)	386 (0.6)	0.99 (0.86-1.14)	1.00 (0.87-1.15)	0.998
**Age (in years)**					
0-11	13650 (10.5)	94 (0.7)	0.99 (0.78-1.27)	0.50 (0.38-0.65)	<0.001
12-17	12093 (9.3)	152 (1.3)	1.85 (1.50-2.28)	1.11 (0.88-1.40)	0.374
18-29	34549 (26.6)	235 (0.7)	1.03 (0.86-1.25)	1.06 (0.87-1.28)	0.567
30-44	30755 (23.6)	209 (0.7)	Ref.	Ref.	-
45-54	18633 (14.3)	71 (0.4)	0.56 (0.42-0.73)	0.65 (0.50-0.86)	0.002
55-64	11534 (8.9)	27 (0.2)	0.34 (0.23-0.51)	0.40 (0.27-0.61)	<0.001
65-74	5005 (3.9)	11 (0.2)	0.32 (0.17-0.58)	0.36 (0.19-0.67)	0.001
>=75	3829 (2.9)	10 (0.3)	0.38 (0.20-0.71)	0.26 (0.13-0.51)	<0.001
**County**					
Agder	5425 (4.2)	18 (0.3)	Ref.	-	-
Innlandet	5913 (4.5)	23 (0.4)	1.12 (0.61-2.08)		
Møre og Romsdal	2017 (1.6)	3 (0.1)	0.46 (0.14-1.58)		
Nordland	1648 (1.3)	3 (0.2)	0.55 (0.16-1.88)		
Oslo	36645 (28.2)	343 (0.9)	2.67 (1.66-4.29)		
Rogaland	7493 (5.8)	35 (0.5)	1.42 (0.80-2.51)		
Troms og Finnmark	2424 (1.9)	14 (0.6)	1.81 (0.90-3.65)		
Trøndelag	5411 (4.2)	25 (0.5)	1.38 (0.75-2.53)		
Vestfold og Telemark	8867 (6.8)	36 (0.4)	1.18 (0.67-2.07)		
Vestland	10069 (7.7)	56 (0.6)	1.65 (0.97-2.81)		
Viken	44136 (33.9)	253 (0.6)	1.64 (1.02-2.65)		
**Country of birth**					
Foreign	42958 (33.0)	284 (0.7)	Ref.	-	-
Norway	84979 (65.3)	522 (0.6)	0.94 (0.81-1.09)		
Unknown	2111 (1.6)	3 (0.1)	0.21 (0.07-0.66)		
**Risk group**					
No comorbidity	112273 (86.3)	715 (0.6)	Ref.	Ref.	.
Medium risk comorbidity	16071 (12.4)	83 (0.5)	0.80 (0.63-1.00)	1.19 (0.94-1.51)	0.143
High risk comorbidity	1704 (1.3)	11 (0.7)	0.99 (0.55-1.79)	1.70 (0.92-3.12)	0.088
**Vaccine status**					
Unvaccinated	38068 (29.3)	464 (1.2)	Ref.	Ref.	-
Vaccinated with one dose <21 days earlier	1889 (1.4)	31 (1.6)	0.86 (0.60-1.25)	0.83 (0.57-1.21)	0.329
Vaccinated with one dose >=21 days earlier	73536 (56.5)	262 (0.4)	0.33 (0.28-0.38)	0.29 (0.24-0.35)	<0.001
Maximum of two doses 7-179 days prior	12062 (9.3)	29 (0.2)	0.34 (0.23-0.50)	0.35 (0.24-0.52)	<0.001
Maximum of two doses ≥180 days prior	2936 (2.3)	20 (0.7)	0.76 (0.48-1.18)	0.85 (0.53-1.37)	0.514
Three doses	1557 (1.2)	3 (0.2)	0.41 (0.13-1.27)	0.64 (0.20-2.08)	0.459
**Most recent infection prior to Delta wave**					
Pre-alpha infection	44003 (33.8)	311 (0.7)	Ref.	Ref.	-
Inter-wave pre-alpha/Alpha	31797 (24.4)	212 (0.7)	0.94 (0.79-1.12)	0.85 (0.71-1.01)	0.070
Alpha wave infection	43551 (33.5)	231 (0.5)	0.75 (0.63-0.89)	0.58 (0.49-0.70)	<0.001
Inter-wave Alpha/Delta	10697 (8.2)	55 (0.5)	1.45 (1.08-1.94)	1.04 (0.77-1.41)	0.793

Characteristics of SARS-CoV-2 reinfection cases during the Delta wave, using a 60-day interval between cases. Hazard ratio estimates for reinfection using stratified Cox regression model in Norway 26 February - 31 January 2022. (*n* = 130 048)

^*^Sex, age group, risk group, vaccine status, the most recent infection prior to the Delta wave was included in a multivariate model, stratifying for county of residence and country of birth

Regarding the association between reinfections and sex, we found that during the Omicron wave, women had a slightly increased risk of reinfection compared to men, whereas no significant difference was observed during the Alpha and Delta waves. This finding is consistent with studies from France [4] and Serbia, [6], but not Iceland [5]. However, the number of reinfections during Alpha and Delta waves was small, resulting in lack of power to detect potential differences. Furthermore, the higher risk of reinfections in women during the early Omicron wave was only slight and of limited practical significance. In general, the reinfection rates for Omicron largely followed infection numbers for January 2022, when there was no difference between the number of infected among men and women [36]

Throughout the Alpha, Delta, and Omicron waves, the infection rate among individuals aged 60 years and above remained low compared to teenagers and young adults [21, 37–39]. A similar pattern was observed for reinfection risk, with a reduced risk of reinfection during the Omicron and Delta waves among age groups 44 years or older, compared to the 30–44 year-olds. The 30–44 age group was chosen as a reference as the older age groups and risk groups were prioritized for vaccination and this could affect the risk of infection and subsequent reinfection. Likewise, younger age groups were vaccinated later with no strong vaccine recommendation to vaccinate healthy adolescents <16 years old [22]. The highest risk of reinfection during the early Omicron wave was found among the 12–17 year-olds, which corresponds to the overall infection risk between different age groups at the time [21]. We should note that changes in testing requirements such as mass testing in school was introduced and maintained in several counties during the Alpha, Delta and Omicron waves. This could have influenced the detection of cases and reinfections, including more asymptomatic cases as well, among children and teenagers aged 6-18 years. However, stratifying on individual test activity did not impact the conclusions (Additional file 5). It is possible that the introduction of rapid antigen tests impacted the children subjected to mass testing differently than the general population, and “testing fatigue” could cause a greater reduction in the proportion of positive tests subsequently confirmed by PCR. Studies assessing reinfections during the early Omicron wave in Iceland and France have similarly found a decreased reinfection risk among the older age groups [4, 5] The risk of developing severe disease from SARS-CoV-2 increases with age [40] which might lead to behavioral changes in older individuals resulting in less social contacts than younger individuals. Additionally, older individuals and those with high-risk comorbidities were among the first to be offered vaccines and subsequent booster doses against COVID-19 [22]. Although the model is adjusted for vaccine status, it is possible that there are residual confounding which could explain the decreased reinfection risk among these groups during the Omicron wave.

Throughout the pandemic the proportions of SARS-CoV-2 cases and hospitalizations have been higher among individuals born abroad compared to those born in Norway, with variations observed between countries of origin [12, 41]. Therefore, a reduced risk of reinfections among Norwegian-born individuals could be anticipated. However, this difference was only observed during the Alpha wave, and the slight reduction in risk among Norwegian-born individuals during the early Omicron wave, as seen in the exploratory analysis (Additional file 1), holds limited practical significance. Previous reports attribute the higher infection risk among individuals born abroad to socioeconomic disparities, densely populated areas, cramped living conditions and increased contact with individuals traveling between countries [41]. The impact of these factors may have been more pronounced in the early stages of the pandemic but could have been partially mitigated by public health interventions to increase awareness in these group and by the emergence of more infectious variants later on.

The time since previous infection has been shown to correlate with the risk of reinfections [42]. The lower protection against Omicron among individuals previously infected during earlier waves should be interpreted as an effect of the time since the previous infection, rather than different protection against Omicron conferred by the different variants. Although differences in protection against Omicron conferred by the various variants cannot be entirely excluded, the sequential nature of the waves in this study makes it unsuitable for exploring such differences. In addition to previous infections, vaccines could also contribute to the population’s resistance towards the different variants. Vaccines have previously been shown to be effective against infections and severe outcomes of COVID-19 [19, 31]. There was a clear protective effect of the vaccines against reinfections, however, there did not seem to be a large difference in risk between those receiving two doses and those who received an additional booster.

Despite our efforts, the study has some additional limitations. Using a fixed interval to define reinfections does not consider full recovery or persistent infection. This is especially challenging for surveillance systems during a pandemic with high case load. Defining reinfections based on sequence and variant typing in surveillance systems during this magnitude of cases is daunting, emphasizing the need for a globally agreed-upon definition. Another limitation is that the results were not adjusted for test activity. Age and county adjustments in the multivariable model probably reduced this bias, and a secondary analysis stratifying for test activity did not impact the conclusions (Additional file 5). The introduction of rapid antigen tests, especially towards the end of 2021, could have caused an underestimation of reinfections. However, all individuals with a positive self-administered antigen test were recommended to get a free of charge PCR test, and test activity remained high until the end of January 2022 [12]. We therefore believe that the introduction of self-administered antigen test had little impact on the probability of reporting a positive test, although limited underreporting cannot be completely ruled out towards the end of 2021 and in January 2022. Variations in health behaviors among groups, unaccounted for in this study, could be potential confounders and we did not have information on differences in behavior patterns to assess how these could impact our findings. The results of this study are not only relevant to Norway, but can be generalized with consideration of each country’s test capacity, restrictions and measurements. However, identifying reinfections using a 60 day interval requires a surveillance system registering all tests.

## Conclusion

The emergence of the Omicron variant led to a substantial increase in infections and reinfections in Norway, with the highest risk of detected reinfections observed among teenagers and young adults. The risk of reinfection seemed to follow similar patterns as the risk of first infection. Individuals with previous/first infections during waves at the start of the pandemic had a higher risk of reinfections than those with infected during one of the more recent waves, indicating that post infection waning immunity is an important factor. Vaccination against SARS-CoV-2 was associated with protection against reinfection. Our findings could assist evaluating vaccination polices for people previously infected but further studies are needed to evaluate the impact of multiple vaccine doses and waning immunity.

### Supplementary Information

**Additional file 1.** Exploratory analysis of characteristics of SARS-CoV-2 reinfection during the Omicron wave.

**Additional file 2.** Exploratory analysis of characteristics of SARS-CoV-2 reinfection during the Delta wave.

**Additional file 3.** Exploratory analysis of characteristics of SARS-CoV-2 reinfection during the Alpha wave.

**Additional file 4.** Characteristics of SARS-CoV-2 reinfections during the Alpha wave, using a 60-day interval between cases.

**Additional file 5.** Reinfections during Alpha, Delta and Omicron waves. Stratified on county, birth-country and SARS-CoV-2 tests activity.

## Data Availability

The datasets analyzed during the current study come from the national emergency preparedness registry for COVID-19, housed at the Norwegian Institute of Public Health. The preparedness registry comprises data from a variety of central health registries, national clinical registries and other national administrative registries. Further information on the preparedness registry, including access to data from each data source, is available at https://www.fhi.no/en/id/infectious-diseases/coronavirus/emergency-preparedness-register-for-covid-19/ [15].Further information on the preparedness registry, including access to data from each data source, is available at https://www.fhi.no/en/id/infectious-diseases/coronavirus/emergency-preparedness-register-for-covid-19/ [15].

## Abbreviations

ECDC: The European Center for Disease Control
BeredtC19: Norwegian national preparedness registry for COVID-19
NPR: The Norwegian Patient Registry
KUHR: Norwegian Control and Payment of Health Reimbursements Database
CI: Confidence intervals
aHR: Adjusted hazard ratios
