# Supplementary material for: Frequency and risk of SARS-CoV-2 reinfections in Norway: a nation-wide study, February 2020 to January 2022
Source: BMC Public Health. 2024 Jan 15;24:181. doi: 10.1186/s12889-024-17695-8 (PMC10789014; doi:10.1186/s12889-024-17695-8)
Supplement: Supplementary file 1 — Additional file 1. Exploratory analysis of characteristics of SARS-CoV-2 reinfection during the Omicron wave. [file 12889_2024_17695_MOESM1_ESM.docx]

**Additional file 1: Exploratory analysis of characteristics of SARS-CoV-2 reinfection during the Omicron wave.**

|  | **Hazard ratio Stratified*** | **Stratified aP^*^** | **Odds Ratio** | **Adjusted odds ratio**^†^ | **Adjusted P-value**^†^ |
| --- | --- | --- | --- | --- | --- |
| **Sex** |  |  |  |  |  |
| Male |  |  |  |  |  |
| Female | 1.14 (1.10-1.18) | <0.001 | 1.11 (1.07-1.14) | 1.15 (1.11-1.18) | <0.001 |
| **Age (in years)** |  |  |  |  |  |
| 0-11 | 1.02 (0.96-1.09) | 0.582 | 1.38 (1.31-1.46) | 1.08 (1.01-1.15) | 0.016 |
| 12-17 | 1.62 (1.53-1.71) | <0.001 | 1.97 (1.88-2.06) | 1.83 (1.73-1.93) | <0.001 |
| 18-29 | 1.18 (1.13-1.25) | <0.001 | 1.23 (1.17-1.29) | 1.20 (1.14-1.26) | <0.001 |
| 30-44 |  |  |  |  |  |
| 45-54 | 0.72 (0.67-0.77) | <0.001 | 0.62 (0.58-0.66) | 0.70 (0.65-0.75) | <0.001 |
| 55-64 | 0.40 (0.36-0.45) | <0.001 | 0.32 (0.29-0.36) | 0.39 (0.35-0.44) | <0.001 |
| 65-74 | 0.20 (0.16-0.25) | <0.001 | 0.13 (0.10-0.16) | 0.19 (0.15-0.24) | <0.001 |
| >=75 | 0.10 (0.07-0.15) | <0.001 | 0.08 (0.06-0.11) | 0.11 (0.08-0.16) | <0.001 |
| **County** |  |  |  |  |  |
| Agder |  |  |  |  |  |
| Innlandet | 0.75 (0.66-0.86) | <0.001 | 0.75 (0.66-0.85) | 0.78 (0.68-0.89) | <0.001 |
| Møre og Romsdal | 0.42 (0.34-0.51) | <0.001 | 0.38 (0.31-0.47) | 0.43 (0.35-0.53) | <0.001 |
| Nordland | 0.48 (0.39-0.59) | <0.001 | 0.41 (0.33-0.50) | 0.50 (0.41-0.62) | <0.001 |
| Oslo | 1.50 (1.37-1.64) | <0.001 | 1.64 (1.50-1.80) | 1.60 (1.46-1.76) | <0.001 |
| Rogaland | 0.66 (0.59-0.75) | <0.001 | 0.65 (0.58-0.74) | 0.67 (0.59-0.76) | <0.001 |
| Troms og Finnmark | 0.36 (0.30-0.43) | <0.001 | 0.31 (0.26-0.37) | 0.37 (0.31-0.45) | <0.001 |
| Trøndelag | 0.83 (0.74-0.93) | 0.002 | 0.80 (0.71-0.90) | 0.90 (0.80-1.01) | 0.065 |
| Vestfold og Telemark | 0.99 (0.89-1.11) | 0.908 | 0.98 (0.88-1.10) | 0.97 (0.87-1.09) | 0.621 |
| Vestland | 0.77 (0.69-0.86) | <0.001 | 0.77 (0.69-0.86) | 0.79 (0.70-0.88) | <0.001 |
| Viken | 1.27 (1.16-1.39) | <0.001 | 1.35 (1.23-1.48) | 1.32 (1.20-1.45) | <0.001 |
| **Country of birth** |  |  |  |  |  |
| Foreign |  |  |  |  |  |
| Norway | 0.95 (0.91-0.98) | 0.005 | 0.95 (0.92-0.99) | 0.93 (0.89-0.97) | <0.001 |
| Unknown | 0.53 (0.34-0.83) | 0.006 | 0.07 (0.05-0.12) | 0.46 (0.29-0.72) | 0.001 |
| **Risk group** |  |  |  |  |  |
| No comorbidity |  |  |  |  |  |
| Medium risk comorbidity | 1.07 (1.01-1.13) | 0.028 | 0.69 (0.65-0.73) | 1.10 (1.03-1.16) | 0.003 |
| High risk comorbidity | 0.78 (0.61-0.99) | 0.037 | 0.34 (0.27-0.43) | 0.77 (0.61-0.98) | 0.036 |
| **Vaccine status** |  |  |  |  |  |
| Unvaccinated |  |  |  |  |  |
| Vaccinated with one dose <21 days earlier | 0.38 (0.31-0.46) | <0.001 | 0.52 (0.42-0.63) | 0.43 (0.35-0.53) | <0.001 |
| One dose | 0.72 (0.69-0.75) | <0.001 | 0.82 (0.79-0.85) | 0.69 (0.66-0.72) | <0.001 |
| Maximum of two doses 7-179 days prior | 0.40 (0.38-0.42) | <0.001 | 0.36 (0.34-0.38) | 0.43 (0.40-0.45) | <0.001 |
| Maximum of two doses ≥180 days prior | 0.35 (0.31-0.40) | <0.001 | 0.14 (0.12-0.16) | 0.34 (0.30-0.38) | <0.001 |
| Three doses | 0.40 (0.33-0.47) | <0.001 | 0.22 (0.18-0.26) | 0.43 (0.36-0.52) | <0.001 |
| **Most recent infection prior to Omicron wave** |  |  |  |  |  |
| Pre-alpha infection |  |  |  |  |  |
| Inter-wave pre-alpha/Alpha | 0.93 (0.88-0.98) | 0.006 | 1.12 (1.06-1.18) | 0.93 (0.88-0.98) | 0.007 |
| Alpha wave infection | 0.87 (0.83-0.91) | <0.001 | 1.14 (1.09-1.20) | 0.88 (0.83-0.92) | <0.001 |
| Inter-wave Alpha/Delta | 0.84 (0.77-0.91) | <0.001 | 0.94 (0.86-1.02) | 0.84 (0.77-0.91) | <0.001 |
| Delta wave infection | 0.58 (0.56-0.61) | <0.001 | 0.60 (0.57-0.63) | 0.45 (0.43-0.47) | <0.001 |
| Inter-wave Delta/Omikron | - | - | - | - | - |
| Characteristics using a stratified Cox regression model and univariate and multivariate random-effects logit model of SARS-CoV-2 reinfection during the Omicron wave cases using a 60-day interval between cases. (n = 258 107)  *Each variable is included in a multivariate model with the “Most recent infection prior to the Omicron wave”, stratifying for all other variables. For the variable “Most recent infection prior to the Omicron wave”, sex was included in the multivariate model, stratifying for all other variables. †Sex, age group, risk group, vaccine status, the most recent infection prior to the Omicron wave was included as independent variables in a multivariate random-effects logit model | | | | | |
