# Supplementary material for: Frequency and risk of SARS-CoV-2 reinfections in Norway: a nation-wide study, February 2020 to January 2022
Source: BMC Public Health. 2024 Jan 15;24:181. doi: 10.1186/s12889-024-17695-8 (PMC10789014; doi:10.1186/s12889-024-17695-8)
Supplement: Supplementary file 3 — Additional file 3. Exploratory analysis of characteristics of SARS-CoV-2 reinfection during the Alpha wave. [file 12889_2024_17695_MOESM3_ESM.docx]

**Additional file 3: Exploratory analysis of characteristics of SARS-CoV-2 reinfection during the Alpha wave.**

|  | **Hazard ratio Stratified*** | **Stratified aP^*^** | **Odds Ratio** | **Adjusted odds ratio**^†^ | **Adjusted P-value**^†^ |
| --- | --- | --- | --- | --- | --- |
| **Sex** |  |  |  |  |  |
| Male |  |  |  |  |  |
| Female | 1.27 (0.90-1.79) | 0.174 | 1.18 (0.84-1.66) | 1.26 (0.90-1.77) | 0.181 |
| **Age (in years)** |  |  |  |  |  |
| 0-11 | 0.27 (0.08-0.88) | 0.031 | 0.21 (0.06-0.68) | 0.25 (0.08-0.84) | 0.024 |
| 12-17 | 1.13 (0.59-2.15) | 0.720 | 0.95 (0.50-1.78) | 1.07 (0.56-2.05) | 0.830 |
| 18-29 | 1.45 (0.93-2.26) | 0.098 | 1.29 (0.85-1.98) | 1.43 (0.92-2.21) | 0.110 |
| 30-44 |  |  |  |  |  |
| 45-54 | 0.45 (0.22-0.91) | 0.026 | 0.43 (0.21-0.86) | 0.45 (0.22-0.91) | 0.027 |
| 55-64 | 0.68 (0.33-1.40) | 0.298 | 0.63 (0.31-1.26) | 0.70 (0.34-1.43) | 0.324 |
| 65-74 | 1.06 (0.43-2.58) | 0.905 | 0.98 (0.44-2.19) | 1.02 (0.43-2.45) | 0.962 |
| >=75 | 1.15 (0.39-3.43) | 0.797 | 0.97 (0.41-2.29) | 1.15 (0.42-3.10) | 0.788 |
| **County** |  |  |  |  |  |
| Agder |  |  |  |  |  |
| Innlandet | 2.63 (0.29-23.58) | 0.389 | 2.67 (0.30-23.93) | 2.66 (0.30-23.85) | 0.382 |
| Møre og Romsdal | 6.48 (0.67-62.56) | 0.106 | 7.44 (0.77-71.55) | 7.07 (0.73-68.05) | 0.091 |
| Nordland | 8.57 (0.96-76.85) | 0.055 | 8.54 (0.95-76.50) | 8.55 (0.95-76.55) | 0.055 |
| Oslo | 3.76 (0.52-27.40) | 0.192 | 4.24 (0.58-30.83) | 4.01 (0.55-29.22) | 0.170 |
| Rogaland | 5.30 (0.67-41.92) | 0.114 | 5.98 (0.76-47.19) | 5.76 (0.73-45.49) | 0.097 |
| Troms og Finnmark | 5.95 (0.62-57.42) | 0.123 | 5.94 (0.62-57.17) | 5.90 (0.61-56.85) | 0.125 |
| Trøndelag | 4.87 (0.61-39.04) | 0.136 | 5.44 (0.68-43.51) | 5.38 (0.67-43.03) | 0.113 |
| Vestfold og Telemark | 6.05 (0.79-46.57) | 0.084 | 6.47 (0.84-49.77) | 6.21 (0.81-47.76) | 0.080 |
| Vestland | 5.99 (0.80-45.00) | 0.082 | 6.60 (0.88-49.46) | 6.35 (0.84-47.75) | 0.072 |
| Viken | 2.66 (0.36-19.50) | 0.336 | 2.83 (0.39-20.69) | 2.85 (0.39-20.88) | 0.303 |
| **Country of birth** |  |  |  |  |  |
| Foreign |  |  |  |  |  |
| Norway | 0.64 (0.44-0.92) | 0.015 | 0.58 (0.41-0.82) | 0.58 (0.40-0.83) | 0.003 |
| Unknown | 1.66 (0.49-5.59) | 0.417 | 1.05 (0.38-2.89) | 0.98 (0.32-2.99) | 0.966 |
| **Risk group** |  |  |  |  |  |
| No comorbidity |  |  |  |  |  |
| Medium risk comorbidity | 1.46 (0.87-2.43) | 0.150 | 1.18 (0.74-1.88) | 1.55 (0.94-2.55) | 0.087 |
| High risk comorbidity | 4.79 (1.93-11.87) | 0.001 | 3.01 (1.32-6.86) | 4.26 (1.77-10.28) | 0.001 |
| **Vaccine status** |  |  |  |  |  |
| Unvaccinated |  |  |  |  |  |
| Vaccinated with one dose <21 days earlier | 0.42 (0.10-1.78) | 0.237 | 0.34 (0.09-1.39) | 0.34 (0.08-1.37) | 0.128 |
| One dose | 0.57 (0.22-1.52) | 0.265 | 0.38 (0.15-0.92) | 0.34 (0.14-0.86) | 0.023 |
| Maximum of two doses 7-179 days prior | 0.25 (0.05-1.28) | 0.096 | 0.46 (0.11-1.85) | 0.34 (0.08-1.47) | 0.149 |
| **Most recent infection prior to Alpha wave** |  |  |  |  |  |
| Pre-alpha infection |  |  |  |  |  |
| Inter-wave pre-alpha/Alpha | 1.26 (0.89-1.80) | 0.196 | 1.07 (0.76-1.51) | 1.07 (0.75-1.51) | 0.719 |
| Characteristics using a stratified Cox regression model and univariate and multivariate random-effects logit model of SARS-CoV-2 reinfection during the Alpha wave cases using a 60-day interval between cases (n = 75 986).  *Each variable is included in a multivariate model with the “Most recent infection prior to the Alpha wave”, stratifying for all other variables. For the variable “Most recent infection prior to the Alpha wave”, sex was included in the multivariate model, stratifying for all other variables. †Sex, age group, risk group, vaccine status, the most recent infection prior to the Alpha wave was included as independent variables in a multivariate random-effects logit model | | | | | |
