# Supplementary material for: Frequency and risk of SARS-CoV-2 reinfections in Norway: a nation-wide study, February 2020 to January 2022
Source: BMC Public Health. 2024 Jan 15;24:181. doi: 10.1186/s12889-024-17695-8 (PMC10789014; doi:10.1186/s12889-024-17695-8)
Supplement: Supplementary file 4 — Additional file 4. Characteristics of SARS-CoV-2 reinfections during the Alpha wave, using a 60-day interval between cases. [file 12889_2024_17695_MOESM4_ESM.docx]

**Additional file 4: Characteristics of SARS-CoV-2 reinfections during the Alpha wave, using a 60-day interval between cases.**

|  | **Previously infected individuals at the start of the Alpha wave n (%)** | **Reinfections n(%)** | **Hazard ratio** | **Adjusted Hazard ratio^*^** | **Adjusted P-value^*^** |
| --- | --- | --- | --- | --- | --- |
| **Sex** |  |  |  |  |  |
| Male | 39233 (51.6) | 64 (0.2) |  |  |  |
| Female | 36753 (48.4) | 71 (0.2) | 1.18 (0.84-1.65) | 1.22 (0.87-1.72) | 0.245 |
| **Age (in years)** |  |  |  |  |  |
| 0-11 | 6901 (9.1) | 3 (0.0) | 0.22 (0.07-0.71) | 0.28 (0.08-0.91) | 0.034 |
| 12-17 | 6658 (8.8) | 13 (0.2) | 0.97 (0.52-1.82) | 1.16 (0.61-2.21) | 0.661 |
| 18-29 | 18076 (23.8) | 48 (0.3) | 1.29 (0.84-1.97) | 1.45 (0.93-2.24) | 0.098 |
| 30-44 | 18563 (24.4) | 38 (0.2) |  |  |  |
| 45-54 | 11413 (15.0) | 10 (0.1) | 0.43 (0.21-0.85) | 0.43 (0.22-0.88) | 0.020 |
| 55-64 | 7795 (10.3) | 10 (0.1) | 0.62 (0.31-1.24) | 0.65 (0.32-1.34) | 0.244 |
| 65-74 | 3512 (4.6) | 7 (0.2) | 0.95 (0.42-2.12) | 0.95 (0.39-2.28) | 0.901 |
| >=75 | 3068 (4.0) | 6 (0.2) | 0.92 (0.39-2.18) | 1.12 (0.40-3.09) | 0.833 |
| **County** |  |  |  |  |  |
| Agder | 2345 (3.1) | 1 (0.0) |  |  |  |
| Innlandet | 3507 (4.6) | 4 (0.1) | 2.44 (0.27-21.82) |  |  |
| Møre og Romsdal | 947 (1.3) | 3 (0.3) | 6.79 (0.71-65.24) |  |  |
| Nordland | 1096 (1.4) | 4 (0.4) | 8.28 (0.93-74.11) |  |  |
| Oslo | 22640 (29.8) | 41 (0.2) | 4.13 (0.57-30.04) |  |  |
| Rogaland | 3528 (4.6) | 9 (0.3) | 5.59 (0.71-44.09) |  |  |
| Troms og Finnmark | 1185 (1.6) | 3 (0.3) | 5.57 (0.58-53.51) |  |  |
| Trøndelag | 3449 (4.5) | 8 (0.2) | 4.88 (0.61-39.05) |  |  |
| Vestfold og Telemark | 4355 (5.7) | 12 (0.3) | 6.43 (0.84-49.44) |  |  |
| Vestland | 6392 (8.4) | 18 (0.3) | 6.05 (0.81-45.30) |  |  |
| Viken | 26542 (34.9) | 32 (0.1) | 2.73 (0.37-19.96) |  |  |
| **Country of birth** |  |  |  |  |  |
| Foreign | 25546 (33.6) | 62 (0.2) |  |  |  |
| Norway | 48870 (64.3) | 69 (0.1) | 0.58 (0.41-0.82) |  |  |
| Unknown | 1570 (2.1) | 4 (0.3) | 1.01 (0.37-2.78) |  |  |
| **Risk group** |  |  |  |  |  |
| No comorbidity | 64205 (84.5) | 108 (0.2) |  |  |  |
| Medium risk comorbidity | 10594 (13.9) | 21 (0.2) | 1.16 (0.73-1.86) | 1.50 (0.91-2.47) | 0.112 |
| High risk comorbidity | 1187 (1.6) | 6 (0.5) | 2.95 (1.30-6.71) | 4.03 (1.67-9.74) | 0.002 |
| **Vaccine status** |  |  |  |  |  |
| Unvaccinated | 56735 (74.7) | 126 (0.2) |  |  |  |
| Vaccinated with one dose <21 days earlier | 3980 (5.2) | 2 (0.1) | 0.37 (0.09-1.48) | 0.37 (0.09-1.52) | 0.167 |
| One dose | 12323 (16.2) | 5 (0.0) | 0.56 (0.23-1.40) | 0.54 (0.21-1.38) | 0.195 |
| Maximum of two doses 7-179 days prior | 2948 (3.9) | 2 (0.1) | 0.49 (0.12-2.00) | 0.40 (0.09-1.78) | 0.229 |
| **Most recent infection prior to Alpha wave** |  |  |  |  |  |
| Pre-alpha infection | 44108 (58.0) | 76 (0.2) |  |  |  |
| Inter-wave pre-alpha/Alpha | 31878 (42.0) | 59 (0.2) | 1.33 (0.94-1.88) | 1.31 (0.92-1.86) | 0.137 |
| Characteristics of SARS-CoV-2 reinfection cases during the Alpha wave, using a 60-day interval between cases. Hazard ratio estimates for reinfection using stratified Cox regression model in Norway 26 February - 31 January 2022 (n = 75 986).  *Sex, age group, risk group, vaccine status, the most recent infection prior to the Alpha wave was included in a multivariate model, stratifying for county of residence and country of birth | | | | | |
