# Supplementary material for: Frequency and risk of SARS-CoV-2 reinfections in Norway: a nation-wide study, February 2020 to January 2022
Source: BMC Public Health. 2024 Jan 15;24:181. doi: 10.1186/s12889-024-17695-8 (PMC10789014; doi:10.1186/s12889-024-17695-8)
Supplement: Supplementary file 5 — Additional file 5. Reinfections during Alpha, Delta and Omicron waves. Stratified on county, birth-country and SARS-CoV-2 tests activity. [file 12889_2024_17695_MOESM5_ESM.docx]

**Additional file 5:**  Reinfections during Alpha, Delta and Omicron waves. Stratified on county, birth-country and SARS-CoV-2 tests activity.

|  | **Alpha** | | **Delta** | | **Omicron** | |
| --- | --- | --- | --- | --- | --- | --- |
|  | **Adjusted Hazard ratio^*^** | **Adjusted P-value^*^** | **Adjusted Hazard ratio^*^** | **Adjusted P-value^*^** | **Adjusted Hazard ratio^*^** | **Adjusted P-value^*^** |
| **Sex** |  |  |  |  |  |  |
| Male | Ref. | Ref. | Ref. | Ref. | Ref. | Ref. |
| Female | 1.20 (0.85-1.70) | 0.294 | 0.97 (0.85-1.12) | 0.689 | 1.12 (1.09-1.16) | <0.001 |
| **Age (in years)** |  |  |  |  |  |  |
| 0-11 | 0.34 (0.10-1.11) | 0.074 | 0.56 (0.42-0.74) | <0.001 | 1.14 (1.07-1.22) | <0.001 |
| 12-17 | 1.22 (0.64-2.35) | 0.548 | 1.15 (0.91-1.46) | 0.240 | 1.64 (1.55-1.73) | <0.001 |
| 18-29 | 1.40 (0.89-2.18) | 0.141 | 1.02 (0.84-1.23) | 0.876 | 1.12 (1.06-1.17) | <0.001 |
| 30-44 | Ref. | Ref. | Ref. | Ref. | Ref. | Ref. |
| 45-54 | 0.46 (0.23-0.94) | 0.033 | 0.66 (0.50-0.86) | 0.003 | 0.75 (0.70-0.80) | <0.001 |
| 55-64 | 0.72 (0.35-1.48) | 0.370 | 0.44 (0.29-0.66) | <0.001 | 0.44 (0.39-0.49) | <0.001 |
| 65-74 | 1.07 (0.43-2.69) | 0.882 | 0.39 (0.21-0.74) | 0.004 | 0.23 (0.18-0.30) | <0.001 |
| >=75 | 1.16 (0.38-3.55) | 0.797 | 0.29 (0.15-0.57) | <0.001 | 0.11 (0.08-0.16) | <0.001 |
| **Risk group** |  |  |  |  |  |  |
| No comorbidity | Ref. | Ref. | Ref. | Ref. | Ref. | Ref. |
| Medium risk comorbidity | 1.35 (0.81-2.26) | 0.255 | 1.16 (0.92-1.47) | 0.211 | 1.06 (1.00-1.13) | 0.046 |
| High risk comorbidity | 3.69 (1.46-9.34) | 0.006 | 1.59 (0.86-2.93) | 0.139 | 0.73 (0.57-0.92) | 0.009 |
| **Vaccine status** |  |  |  |  |  |  |
| Unvaccinated | Ref. | Ref. | Ref. | Ref. | Ref. | Ref. |
| Vaccinated with one dose <21 days earlier | 0.34 (0.08-1.40) | 0.135 | 0.83 (0.57-1.21) | 0.327 | 0.37 (0.30-0.45) | <0.001 |
| Vaccinated with one dose >=21 days earlier | 0.26 (0.08-0.87) | 0.029 | 0.29 (0.24-0.35) | <0.001 | 0.70 (0.67-0.73) | <0.001 |
| Maximum of two doses 7-179 days prior | 0.29 (0.06-1.39) | 0.122 | 0.33 (0.22-0.49) | <0.001 | 0.38 (0.36-0.40) | <0.001 |
| Maximum of two doses ≥180 days prior |  |  | 0.76 (0.47-1.23) | 0.260 | 0.34 (0.30-0.38) | <0.001 |
| Three doses |  |  | 0.46 (0.14-1.55) | 0.212 | 0.36 (0.30-0.43) | <0.001 |
| **Most recent infection prior to Alpha wave** |  |  |  |  |  |  |
| Pre-alpha infection | Ref. | Ref. | Ref. | Ref. | Ref. | Ref. |
| Inter-wave pre-alpha/Alpha | 1.17 (0.82-1.68) | 0.386 | 0.86 (0.72-1.03) | 0.092 | 0.93 (0.88-0.98) | 0.009 |
| Alpha wave infection |  |  | 0.57 (0.48-0.68) | <0.001 | 0.84 (0.80-0.89) | <0.001 |
| Inter-wave Alpha/Delta |  |  | 0.88 (0.65-1.20) | 0.427 | 0.77 (0.70-0.83) | <0.001 |
| Delta wave infection |  |  |  |  | 0.53 (0.50-0.55) | <0.001 |
| Sociodemographic characteristics of SARS-CoV-2 reinfection cases during the Alpha, Delta and Omicron wave, using a 60-day interval between cases. Hazard ratio estimates for reinfection using stratified Cox regression model in Norway 26 February - 31 January 2022 (n = 75 986, 130 048, 258 107 for the respective waves). | | | | | | |
| *Sex, age group, risk group, vaccine status, the most recent infection prior to the Alpha wave and time since last infection was included in a multivariate model, stratifying for county of residence, country of birth and individual number of registered Sars-CoV-2 tests | | | | | | |
